# Supplementary material for: A Community-Based Intervention in Middle Schools in Spain to Improve HPV Vaccination Acceptance: A “Pill of Knowledge” Approach
Source: Vaccines (Basel). 2025 Dec 24;14(1):22. doi: 10.3390/vaccines14010022 (PMC12846381; doi:10.3390/vaccines14010022)
Supplement: Supplementary file 1 [file vaccines-14-00022-s001.zip › vaccines-4002438-supplementary.pdf]

# 1. QUESTIONNAIRE

**1.1. Age: years**

**1.2. Gender:**

a Female
b Male
c Other

**1.3. Place of residence:**

a Urban (> 5000 inhabitants)
b Rural (<500 inhabitants)
c Semi-urban (500-5000 inhabitants)

**1.4. The information you have about sexual health, do you consider it to be:**

a A lot
b Enough
c Little
d None

**1.5. The sexual information you receive is mainly through: (check only one)**

a Teachers
b Social networks
c Parents
d Siblings
e Friends
f Other (books, TV, video games…)
g You don't receive information

**1.6. Have you ever heard of HPV (Human Papillomavirus)? (circle one number)**

(0 = nothing 5 = a lot) 0 1 2 3 4 5

**1.7. Are you fully vaccinated against the HPV virus? (Full vaccination consists of two doses if you were under 15 years old when you were vaccinated, or three doses if you were 15 years old or older)**

a Yes
b No
c I don't know

**1.8. What level of education do your parents/guardians have? (List the highest level)**

a Primary
b Secondary
c University students
d I don't know.

## PRE-INTERVENTION TEST:

**Read the questions carefully. Circle the answer you think is correct. Don't leave any questions unanswered.**

**1.9. What is HPV?**

a A virus that causes infection in the genitals
b A virus that causes herpes infection
c A virus that causes AIDS infection
d A virus that causes kidney infection
e I don't know

**1.10. Do you think HPV infection affects many people?**

a Yes
b No
c I don't know

**1.11. Do you think HPV infection affects...?**

a Women only
b Men only
c Both
d I don't know.

**1.12. How is HPV transmitted?**

a Through contagion in public restrooms
b Through contact, through hugs
c Through sexual relations
d Through the air
e I don't know

**1.13. Does the condom protect against HPV infection?**

a Always
b Sometimes
c Never
d The condom is only a contraceptive.
e I don't know

**1.14. Where can HPV-related lesions appear?**

a Mouth
b Genitals
c Anus
d All of the above
e I don't know

**1.15. HPV infection:**

a It always gives symptoms
b Sometimes it causes symptoms
c It never gives symptoms
d I don't know

**1.16. HPV infections:**

a They never need treatment
b They only produce genital warts
c They can cause serious illnesses
d I don't know

**1.17. How are HPV diseases prevented?**

- a With frequent washing of hands and genitals
- b With vaccination before the start of sexual relations
- c With the contraceptive pill
- d I don't know

**1.18. Can anyone who has sex get HPV?**

- a Yes
- b No
- c I don't know

**1.19. Does having many sexual partners increase the risk of HPV infection?**

- a Yes
- b No
- c I don't know

**1.20. How is HPV diagnosed?**

- a Through an analysis of vaginal discharge
- b Due to having warts on the genitals
- c The previous two
- d I don't know

**1.21. Where can cancer appear as a result of HPV infection?**

- a Cervix
- b Anus
- c Pharynx
- d All of the above
- e I don't know

**1.22. If I have already been infected with HPV:**

- a I am now protected forever
- b I can get infected more times
- c I wouldn't need to get vaccinated.
- d I'm probably going to get cancer
- e I don't know

**1.23. Is there a vaccine for HPV?**

- a Only in some countries, like Spain
- b It is under study
- c It doesn't exist.
- d I don't know

**1.24. Are HPV vaccines effective in preventing the lesions it can cause? (mark a number)**

(0 = not at all effective 5 = very effective) 0 1 2 3 4 5

**1.25. Are HPV vaccines safe?**

- a Yes, but they may produce some mild symptoms
- b They can cause meningitis
- c They can cause autism
- d I don't know

**1.26. They should get vaccinated:**

- a Only the girls

b Children only
c Both
d I don't know

1.27. **If you are not vaccinated, what should you do if your healthcare providers recommend the vaccine? Would you get vaccinated against HPV?**

a Yes
b No
c I don't know

## POST-INTERVENTION TEST

**Read the questions carefully. Circle the answer you think is correct. Don't leave any questions unanswered.**

**1.28. What is HPV?**

a A virus that causes infection in the genitals
b A virus that causes herpes infection
c A virus that causes AIDS infection
d A virus that causes kidney infection
e I don't know

**1.29. Do you think HPV infection affects many people?**

a Yes
b No
c I don't know

**1.30. Do you think HPV infection affects...?**

a Women only
b Men only
c Both
d I don't know.

**1.31. How is HPV transmitted?**

a Through contagion in public restrooms
b Through contact, through hugs
c Through sexual relations
d Through the air
e I don't know

**1.32. Does the condom protect against HPV infection?**

a Always
b Sometimes
c Never
d The condom is only a contraceptive.
e I don't know

**1.33. Where can HPV-related lesions appear?**

a Mouth
b Genitals
c Anus
d All of the above
e I don't know

**1.34. HPV infection:**

a It always gives symptoms
b Sometimes it causes symptoms
c It never gives symptoms
d I don't know

**1.35. HPV infections:**

a They never need treatment
b They only produce genital warts
c They can cause serious illnesses
d I don't know

**1.36. How are HPV diseases prevented?**

- a With frequent washing of hands and genitals
- b With vaccination before the start of sexual relations
- c With the contraceptive pill
- d I don't know

**1.37. Can anyone who has sex get HPV?**

- a Yes
- b No
- c I don't know

**1.38. Does having many sexual partners increase the risk of HPV infection?**

- a Yes
- b No
- c I don't know

**1.39. How is HPV diagnosed?**

- a Through an analysis of vaginal discharge
- b Due to having warts on the genitals
- c The previous two
- d I don't know

**1.40. Where can cancer appear as a result of HPV infection?**

- a Cervix
- b Anus
- c Pharynx
- d All of the above
- e I don't know

**1.41. If I have already been infected with HPV:**

- a now protected forever
- b I can get infected more times
- c I wouldn't need to get vaccinated.
- d I'm probably going to get cancer
- e I don't know

**1.42. Is there a vaccine for HPV?**

- a Only in some countries, like Spain
- b It is under study
- c It doesn't exist.
- d I don't know

**1.43. Are HPV vaccines effective in preventing the lesions it can cause? (circle one number)**

(0 = not at all effective 5 = very effective) 0 1 2 3 4 5

**1.44. Are HPV vaccines safe?**

- a Yes, but they may produce some mild symptoms
- b They can cause meningitis
- c They can cause autism
- d I don't know

**1.45. They should get vaccinated:**

- a Only the girls

b Children only
c Both
d I don't know

1.46. **If you are not vaccinated, what should you do if your healthcare providers recommend the vaccine? Would you get vaccinated against HPV?**

a Yes
b No
c I don't know
